# Expression of CIAPIN1 in human colorectal cancer and its correlation with prognosis

**DOI:** 10.1186/1471-2407-10-477

**Published:** 2010-09-03

**Authors:** Hai Shi, Yi Zhou, Heliang Liu, Changsheng Chen, Shujun Li, Nanlin Li, Xiaohua Li, Xi Zhang, Hongwei Zhang, Weizhong Wang, Qingchuan Zhao

**Affiliations:** 1 State Key Laboratory of Cancer Biology, Department of Gastrointestinal Surgery, Xijing Hospital, The Fourth Military Medical University, Xi'an, China; 2 Department of Anorectal Surgery, Tianjin Union Medicine Centre, Tianjin, China; 3 Department of Urology Surgery, Xijing Hospital, The Fourth Military Medical University, Xi'an, China; 4 Department of Health Statistics, The Fourth Military Medical University, Xi'an, China; 5 Department of Vascular and Endocrine Surgery, Xijing Hospital, The Fourth Military Medical University, Xi'an, China

## Abstract

**Background:**

The cytokine-induced anti-apoptotic molecule (CIAPIN1) had been found to be a differentially-expressed gene involved in a variety of cancers, and it was also considered as a candidate tumour suppressor gene in gastric cancer, renal cancer and liver cancer. However, studies on the role of CIAPIN1 in colorectal cancer were still unavailable. The aim of this study was to determine the prognostic impact of CIAPIN1 in 273 colorectal cancer (CRC) samples and to investigate the CIAPIN1 expression in CRC cell lines after inducing differentiation.

**Methods:**

Immunohistochemical analysis was performed to detect the expression of CIAPIN1 in CRC samples from 273 patients. The relationship between CIAPIN1 expression and patients' characteristics (gender, age, location of cancer, UICC stage, local recurrence and tumour grade factors) was evaluated. In addition, these patients were followed up for five consecutive years to investigate the relationship between CIAPIN1 expression and the prognosis of CRC. We induced the differentiation of the CRC cell lines HT29 and SW480, in order to detect the expression of CIAPIN1 in the process of CRC cells differentiation.

**Results:**

Results indicated that CIAPIN1 was mainly expressed in the cytoplasm and nucleus, and that its expression level in cancer samples was significantly lower than in normal tissues. The Wilcoxon-Mann-Whitney test showed a significant difference in the differential expression of CIAPIN1 in patients with different T and UICC stages, and tumour grade (*P *= 0.0393, 0.0297 and 0.0397, respectively). The Kaplan-Meier survival analysis demonstrated that the survival time of CRC patients with high expression of CIAPIN1 was longer than those with low expression during the 5-year follow up period (*P *= 0.0002). COX regression analysis indicated that low expression of CIAPIN1, cancer stage of > pT1, distant organ metastasis (pM_1_), regional lymph node metastasis (> pN_1_) and local recurrence (yes) were independent, poor prognostic factors of CRC (*P *= 0.012, *P *= 0.032, *P <*0.001, *P <*0.001, *P <*0.001 respectively). Both Western blotting and RT-PCR showed that CIAPIN1 expression was increased with the degree of differentiation of HT29 and SW480 cells.

**Conclusions:**

CIAPIN1 played an important role in the differentiation of CRC cells, and the differential expression of CIAPIN1 in CRC was closely related to prognosis.

## Background

Colorectal cancer (CRC) is one of the three leading causes of cancer-related death among men and women in the world, and there are approximately 1,020,000 new cases and 530,000 deaths worldwide per year [1,2]. It is one of the most common malignant tumours in China. During the past decades, the incidence of CRC had been increasing, and some reports indicated that the mortality of CRC had been increasing as a result of early metastases [3]. Despite curative surgical resection of the primary tumour, 40% to 50% of the patients ultimately die of metastases [4-6]. Tumour growth and metastasis result from a complex cascade of biological processes. Therefore, understanding key factors in these processes is crucial to the design of new treatment modalities. Although many molecular markers, including carcinoembryonic antigen (CEA), have been exploited for detecting CRC, these lack sensitivity and specificity for evaluating the prognosis of CRC patients [7-10]. Thus, there is an urgent demand for research into novel molecular markers that can serve as diagnostic and prognostic markers for CRC.

Cytokine-induced anti-apoptosis molecule 1 (CIAPIN1) originally named anamorsin or V62, was a newly identified apoptosis-related molecule that had been proved to be a mediator of the RAS signalling pathway. It also played a vital role in foetal liver haematopoiesis [11,12]. Previous studies by our group confirmed that CIAPIN1 was widely expressed in adult and foetal tissues, but that the expression levels varied in different tissues. There were high expressions of CIAPIN1 in well-differentiated or metabolically active tissues [12,13]. We speculated that CIAPIN1 might play important physiological functions in the human body.

Our laboratory also indicated a direct correlation between the loss of CIAPIN1 and the proliferation of tumours in human digestive cancer [14,15]. Immunohistochemical studies had shown that there was low expression of CIAPIN1 in gastric cancer, but the expression in the corresponding adjacent tissues and normal tissues was high. Up-regulation of CIAPIN1 expression in gastric cancer cell lines demonstrated that CIAPIN1 might be involved in the regulation of gastric cancer cell proliferation and apoptosis. These findings suggested that the expression of CIAPIN1 was negatively related to cell proliferation; in particular, the proliferation of cancer cells.

In this study, the expression level of CIAPIN1 was detected in 273 samples from patients with CRC. The relationship between expression of CIAPIN1 and survival time during the 5-year follow-up period was evaluated. In addition, we investigated the expression of CIAPIN1 after inducing differentiation in colorectal cancer cell lines. Our study provides explicit evidence of the regulation of CIAPIN1 in CRC cells and indicates that CIAPIN1 may be a prognostic marker in CRC.

## Methods

### Patients and collection of samples

A total of 273 patients with primary CRC, who underwent surgery at the Department of Anorectal Surgery at Tianjin Union Medicine Centre, were recruited for this study from 2000 to 2003. The mean age was 58 years (range: 23~82 years) with 136 women and 137 men. Cancer tissues, along with normal tissues that were at least 5 cm away from the cancer, were obtained from the patients. Western blot analysis was performed on fresh samples from 40 CRC patients. All 273 patients' survival information of 60 month postoperative follow-up was received by telephone and mail. The median follow-up period was 39.7 months (range: 10~65 months). Patients' characteristics, such as gender, age, location of the tumour, UICC stage, local recurrence and tumour stage factors, were obtained from the medical records. Patient characteristics are summarised in Table 1. All resection samples were confirmed to be CRC by clinical pathology. All the patients were staged based on the UICC staging system. Of the 273 patients, 24 (8.8%) had T1-stage, 33 (12.1%) had T2-stage, 182 (66.7%) had T3-stage, and 34 (12.4%) had T4-stage CRC. Tissues were fixed in 10% formaldehyde, embedded in paraffin, cut into 4- μm sections, and mounted on slides. All patients gave informed consent to use excess pathological specimens for research purposes. The protocols used in the study were approved by the hospital's Protection of Human Subjects Committee. The use of human tissues in this study was approved by the institutional review board of the Fourth Military Medical University and was done in accordance with international guidelines. Of the 273 patients, 23 (8.4%, some CRC patients with stage IV of UICC) received neoadjuvant chemotherapy, 242 (88.6%, CRC patients with stage IIB, IIC, III and IV of UICC) underwent surgery alone and received subsequent chemotherapy, and 31 (11.4%, CRC patients with stage I and IIA of UICC) only received surgical treatment.

**Table 1:** Correlation between clinicopathological characteristics and CIAPIN1 expression

	CIAPIN1 (-/+)	CIAPIN1 (++)	CIAPIN1 (+++)	*P*
**Age**				0.4756
**≤75**	111(62.7)	41(23.2)	25(14.1)	
** > 75**	54(56.3)	31(32.2)	11(11.5)	
**Gender**				0.1939
**Male**	77(56.2)	41(22.9)	19(13.9)	
**Female**	88(64.7)	31(22.8)	17(12.5)	
**Location**				0.5037
**Right**	44(66.7)	14(21.2)	8(12.1)	
**Left**	25(50.0)	19(38.0)	6(12.0)	
**Rectum**	54(60.7)	23(25.8)	12(13.5)	
**Sigmoid**	42(61.8)	16(23.5)	10(14.7)	
**Local recurrence**				0.1914
**Yes**	53(65.4)	21(25.9)	7(8.6)	
**No**	112(60.4)	51(26.4)	29(13.2)	
**pT**				0.0393
**pT_1_**	8(33.3)	10(41.7)	6(25.0)	
**pT_2_**	20(60.6)	9(27.3)	4(12.1)	
**pT_3_**	114(62.6)	48(24.4)	20(11.0)	
**pT_4_**	23(67.7)	5(14.7)	6(17.6)	
**pN**				0.2832
**pN_0_**	85(57.4)	42(28.4)	21(14.2)	
**pN_1_**	80(64.0)	30(24.0)	15(12.0)	
**pM**				0.9502
**pM_0_**	135(60.0)	63(28.0)	27(12.0)	
**pM_1_**	30(62.5)	9(18.8)	9(18.8)	
**L stage**				0.1689
**L_0_**	77(56.6)	38(27.9)	21(15.4)	
**L_1_**	88(64.2)	34(24.8)	15(10.9)	
**V stage**				0.9732
**V_0_**	63(60.0)	27(25.7)	15(14.3)	
**V_1_**	57(61.3)	24(25.8)	12(12.9)	
**V_2_**	45(60.0)	21(28.0)	9(12.0)	
**UICC**				0.0297
**I+II**	71(53.8)	40(30.3)	21(15.9)	
**III+IV**	94(66.7)	32(22.7)	15(10.6)	
**Tumour grade**				0.0397
**Well**	51(75.0)	8(11.8)	9(13.2)	
**Moderately**	92(54.4)	53(31.4)	24(14.2)	
**Poorly**	22(61.1)	11(30.6)	3(8.3)	

### Immunohistochemical staining [12,13]

The cancer and normal tissues from 273 patients were embedded in paraffin and cut into sections for immunohistochemical analysis. Immunohistochemistry was performed using the Histostain-Plus SP kit, which offers superior sensitivity. Briefly, the sections were deparaffinised with xylene and rehydrated through gradient ethanol immersion. Endogenous peroxidase activity was quenched by 0.3% (v/v) hydrogen peroxide in methanol for 20 min, followed by three 5-min washes with PBS. The sections were then blocked with 10% (v/v) normal goat serum in PBS for 1 hr, followed by overnight incubation at 4°C with the anti-CIAPIN1 antibody diluted (1:200, initial concentration 1.8 mg/ml; Mouse anti-CIAPIN1 MAb was developed by our laboratory) [12,13] in PBS containing 3% (wt/vol) BSA. A negative control was performed by replacing the primary antibody with pre-immune mouse serum. After three 5 min washes with PBS containing 0.02% (v/v) Tween-20 (PBST), the sections were treated with biotinylated goat anti-mouse antibody for 20 min at room temperature, followed by three additional 5 min washes with PBST. Then, the specimens were incubated with streptavidin-horseradish peroxidase for 20 min at room temperature followed by repeated washes, as described above. Reaction product was visualised with DAB at room temperature for 5 min. Sections were counterstained with haematoxylin for 30 sec and rinsed with tap water, immediately dehydrated by sequential immersion in gradient ethanol and xylene and were then mounted with Permount onto coverslips. Images were obtained under a light microscope (Olympus BX51; Olympus, Japan) equipped with a DP70 digital camera.

### Immunohistochemical analysis

Sections without the primary antibody were used as negative controls. Clear cell renal cell carcinoma samples that previously showed immunoreactivity to the CIAPIN1 antibody were used as positive controls to confirm CIAPIN1expression. The slides were evaluated independently by two pathologists who were blinded to the study [14-16]. Any disagreement was resolved by consensus after joint review. Expression of CIAPIN1 was evaluated as the percentage of positive cells and staining intensity as previously described. The percentage of positive cells was evaluated quantitatively and scored as 0 for staining of ≤1% of total cells counted, 1 for staining of 2~25%, 2 for staining of 26~50%, 3 for staining of 51~75%, and 4 for staining of >75% of the cells examined. Intensity was graded as follows: 0, no signal; 1, weak; 2, moderate; and 3, strong staining. A total 'staining score' of 0~12 was calculated and graded as negative (-, score 0~1), weak (+, score 2~4), moderate (++, score 5~8), or strong (+++, score 9~12) [16,17].

### Statistical analysis

The relationship between CIAPIN1 expression levels and clinicopathological factors was analysed using the Wilcoxon-Mann-Whitney test. The overall survival time of CRC patients was defined as the time from the surgery to death due to cancer. The Kaplan-Meier method was used to determine the cumulative probability of survival, and data were analysed with the log-rank test. Univariate and multivariate statistical analyses were done using the Cox regression model to investigate the effects of patients' characteristics (CIAPIN1 status, gender, age, location, extent of primary tumour, nodal status, metastasis and histological grade) on overall survival. A score was assigned to each variable for the Cox regression analysis [17,18]. A value of *P *< 0.05 was considered statistically significant.

Additionally, receiver operator characteristic (ROC) curves were constructed for expression of CIAPIN1 as a predictor for 'patient death' and 'cancer local recurrence'. The ROC curve determines how effective a predicted risk value is at discriminating a bivariable outcome (in this case 'death' or 'no death', or 'local recurrence' or 'non- local recurrence') by constructing a graph with sensitivity on the Y-axis and one specificity on the X-axis. It depicts the inverse relationship between sensitivity and specificity.

### Western blotting

Equal amounts of total proteins were loaded on 12% SDS PAGE and electroblotted onto a nitrocellulose membrane. Non-specific binding was blocked with 5% non-fat milk in PBS for 1 hr at room temperature. Then, the membrane was incubated with anti-CIAPIN1 MAb (1:400) overnight at 4°C, rinsed with TBST three times and then incubated with HRP-labelled goat anti-mouse IgG for 1 hr. After three washes with TBST, the bands were developed with the enhanced chemiluminescence reagent for 5 min [14].

### Reverse transcription-PCR analysis

Total RNA was extracted using TRIzol reagent (Invitrogen, Carlsbad, CA) according to the manufacturer's protocol. Two micrograms of RNA were used for reverse transcription. The polymerase chain reaction (PCR) primers used were as follows: for CIAPIN1, 5'-CGG AAT TCATGG CAG ATT TTG GGA TCT C-3' (forward), 5'-GGT CGA CCT AGG CAT CAA GAT TGC TAT C-3' (reverse) and for β-actin, 5'-ATG ATATCG CCG CGC TCG TC-3' (forward), 5'-CGC TCG GTG AGG ATC TTC A-3'(reverse). PCR products were separated on a 1% agarose gel, visualised and photographed under ultraviolet light [14].

### Cell culture

The human CRC cell lines LoVo, CoLo205, HCT116, HT-29, SW620, and SW480 were obtained from the American Type Culture Collection (Rockville, MD, USA) and maintained as recommended. Cells were cultured in DMEM with 2 mM l-glutamine and Earle's balanced salt solution (BSS) adjusted to contain 1.5 g/L sodium bicarbonate, 0.1 mM nonessential amino acids, and 1.0 mM sodium pyruvate. All culture fluid was supplemented with 10% FCS. All cells were cultured with 5% CO_2 _at 37°C in a humidified chamber [17,18].

### Induction of cell differentiation [18,19]

The human colon carcinoma cell lines HT29 and SW480 were cultured in L-15 medium (Sigma-Aldrich). The media contained 10% foetal bovine serum (Atlanta Biologicals), and cells were cultured at 37°C in 5% CO_2_. Differentiation was induced by treatment with 2 mM sodium butyrate (Sigma Chemical) for 6 d, and cells were harvested each day from the beginning of treatment to the sixth day. The differentiation stage was assessed by transmission electron microscopy (TEM; for changes in cell structure and architecture) and degree of alkaline phosphatase (AP) activity. After removal of the culture medium, the attached cells were scraped into ice-cold PBS, centrifuged at 4000 × g, resuspended in PBS, and sonicated with an ultrasonic cell disrupter. Cellular debris was pelleted by centrifugation, and supernatants were transferred to new tubes and stored at -70°C until measurement. AP activity was measured according to the manufacturer's instructions using *p*-nitro-phenylphosphate as the substrate (Merck, Darmstadt, Germany) and calculated in units per milligram protein (U/mg prot). Protein content was determined using a BCA™ Protein Assay Kit.

## Results

### Expression of CIAPIN 1 in Human Colorectal Cancer and Its Correlation With Prognosis

**Figure 1:**
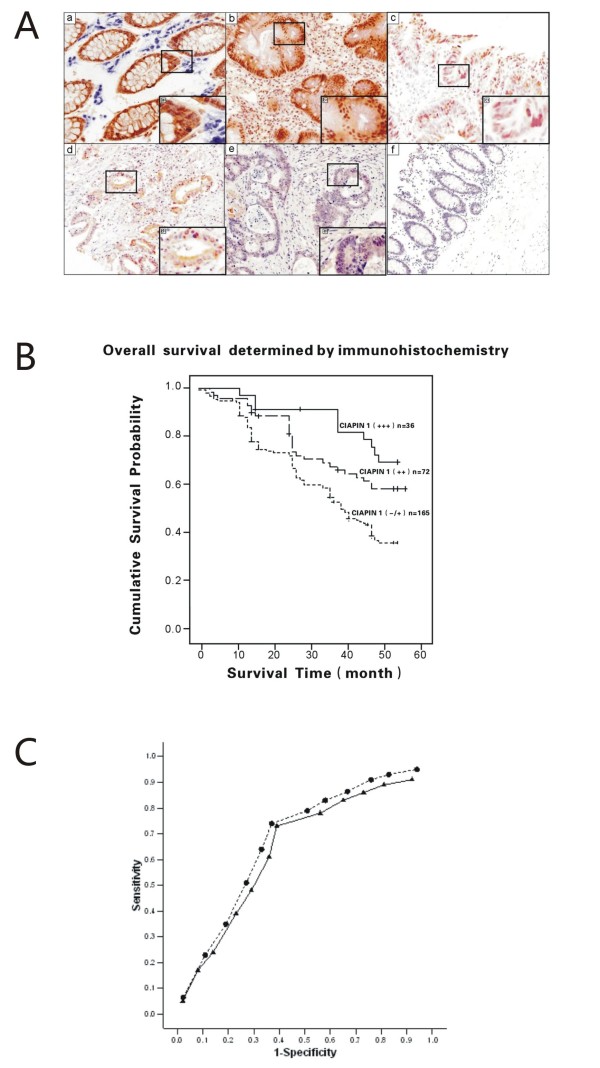
**(A) Immunohistochemical staining of CIAPIN1 in CRC tissues**. (a) Normal colonic epithelium, strong staining (+++) was observed in the normal colon epithelial tissues, mainly in the epithelial cells, and no evidence of expression of CIAPIN1 was noted in cells of the germinal layer (×200). (b) Gland hyperplasia of colonic mucous membrane, strong staining (+++) of CIAPIN1 was observed in dysplasia colonic epithelial tissues, there was no significant difference in the expression level of CIAPIN1 protein between normal and dysplasia colon tissues (×200). (c) Well-differentiated adenocarcinoma of colon, intermediate staining (++) of CIAPIN1 in CRC tissues with high differentiation mainly in the epithelial tissues (×200). (d) Moderately differentiated adenocarcinoma of colon, weak staining (+) of CIAPIN1 in CRC tissues with moderately differentiation, mainly in the epithelial tissues (×200). (e) Poorly differentiated adenocarcinoma of colon, negative staining (-) of CIAPIN1 in CRC tissues with poorly differentiation (×200). (f) Negative control slides using anti-His as the primary antibody. (B) Overall survival of patients determined by the immunoreactivity of CIAPIN1. Overall survival analysis using the Kaplan-Meyer method revealed that CRC patients with relatively high expression of CIAPIN1 had a more favourable prognosis compared to those with low expression (*P *= 0.0002). (C) Receiver operating characteristic (ROC) curve for non-local recurrence (circles) within 5 years and survival (triangles) after 5 years.

The expression of CIAPIN1 by immunoreactivity with the specific MAb was generally localised in both the cytoplasm and the nucleus of colonic epithelial tissues Fig. 1[A(a-e)]. CIAPIN1-positive expression in CRC was 47.25% (129/273), significantly lower than 79.85% (218/273) in the unaffected tissue adjacent to the tumour (*P <*0.01). CIAPIN1 protein expression in CRC was significantly decreased compared with that in normal colonic epithelial tissues. In addition, its expression level decreased from well-differentiated to poorly differentiated tumours Fig. 1[A(c-e)]. However, there was no significant difference in the expression level of CIAPIN1 protein between normal and gland hyperplasia of colonic mucous membrane tissues Fig. 1[A(a-b)]. As shown in Fig. 1A and Table 1, 273 CRC patients were subdivided into the following three subgroups based on the expression levels of CIAPIN1, 165 with none to weak expression (60.4%; -/+), 72 with moderate to locally strong expression (26.4%; ++), and 36 with strong expression (13.2%; +++).

The correlation between expression level of CIAPIN1 and patients' characteristics, such as gender, age, location of cancer, UICC stage, local recurrence and tumour stage factors was investigated. CIAPIN1 protein expression correlated with having a primary tumour (pT), tumour stage (UICC), and histological grade (Table 1; *P *= 0.0393, 0.0297, and 0.0397, respectively). No correlation was observed between CIAPIN1 expression and age, gender, location, local recurrence, L stage, V stage, pN and pM. The overall survival analysis using the Kaplan-Meyer method revealed that the prognosis of CRC patients with high or moderate CIAPIN1 expression was significantly better than those with no or weak CIAPIN1 expression, and high expression was better than moderate expression (Fig. 1(B); *P *= 0.0002).

The ROC curve for non-local recurrence within 5 years (Fig. 1(C)) showed that the score 7 of CIAPIN1 expression level provided maximal sensitivity (0.63) and specificity (0.74). Similarly, the curve for survival after 5 years (Fig. 1(C)) showed that the score 6 of CIAPIN1 expression level provided a maximal sensitivity (0.61) and specificity (0.73). The area under the curve for non-local recurrence was 0.74 and for survival was 0.71. This shows that the score of CIAPIN1 expression level did predict non-local recurrence and survival.

Both univariate and multivariate analyses showed that primary tumour (pT_3_/pT_4_), regional lymph node metastasis (pN_1_), distant tumour metastasis (pM_1_), local recurrence (yes) and low-expression CIAPIN1 (-/+) were independent, poor prognostic factors of CRC (Table 2 and Table 3); However, age (> 75 years), gender (male), location (rectum or sigmoid), adjuvant therapy (yes) and tumour grade (> G2) were not related to the prognosis of CRC (Table 2 and Table 3).

**Table 2:** Cox univariate analysis

Variables	Wald chi-square	df	*P*
Age	2.587	1	0.108
Gender	2.798	1	0.094
Location	0.462	1	0.497
Neoadjuvant therapy	2.474	1	0.116
Local recurrence	5.549	1	0.018
pT	10.861	1	0.001
pN	7.704	1	0.006
pM	14.783	1	< 0.001
L stage	1.431	1	0.232
V stage	2.128	1	0.145
Tumour grade	3.293	1	0.070
CIAPIN1	6.468	1	0.011

Variables (Location, pT, Tumour grade, V satge, CIAPIN1,) in table 2 were adjusted for binary variables

**Table 3:** Cox multivariate analysis

Variables	Risk ratio (95% confidence interval)	*P*
Age (>**7**5 y)	1.100 (0.714-1.694)	0.665
Gender (male)	1.410 (0.712-2.794)	0.324
Location (Rectum or Sigmoid)	0.753(0.512-1.106)	0.149
Neoadjuvant therapy (Yes)	0.661(0.329-1.328)	0.245
Local recurrence (Yes)	2.181(1.382-3.441)	< 0.001
Primary tumour (pT_3 _or pT_4_)	1.697 (1.060-2.718)	0.028
Regional lymph node metastasis (pN_1_)	2.307 (1.434-3.710)	0.001
Distant metastasis (pM_1_)	2.504 (1.555-4.030)	< 0.001
L stage (L_1_)	1.199(0.845-1.700)	0.309
V stage (V_1 _or V_2_)	0.811(0.573-1.148)	0.237
Tumour grade (> G2)	0.723 (0.447-1.170)	0.186
CIAPIN1 (++ or +++)	0.263 (0.114-0.608)	0.002

CIAPIN1: Cytokine-induced anti-apoptotic molecule

Values are mean ± SD, n = 3. CIAPIN1: Cytokine-induced anti-apoptotic molecule

### Analysis of CIAPIN1 Expression by Western Blot

**Figure 2:**
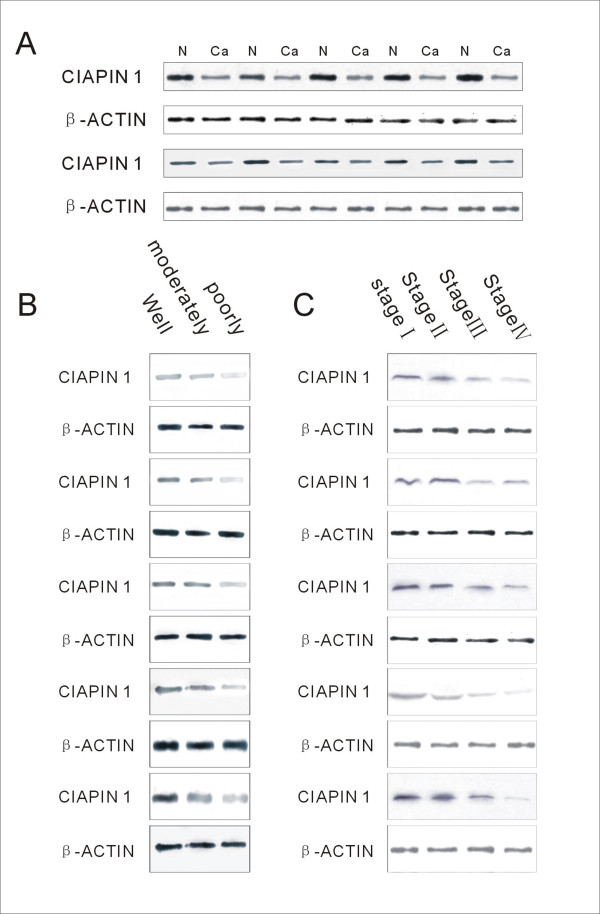
**Detection of CIAPIN1 expression in freshly obtained CRC tissues Western blot analysis**. (A) CIAPIN1 expression level in cancerous and adjacent normal tissues from the same patients. (B) CIAPIN1 expression level in poorly, moderately, and well-differentiated cancer tissues. (C) CIAPIN1 expression in cancer tissues with different TNM stages. β-Actin staining was used as a control for equal protein loading.

To accurately quantify CIAPIN1 expression level changes in colorectal carcinomas, we performed Western blot analysis in 40 CRC tissues and matched normal tissues. Among the 40 clinical specimens, as expected, 28 (70.0%) samples of CRC tissues showed lower expression than that in adjacent normal tissues; 7 (18.5%) samples of CRC tissues showed higher expression than that in adjacent normal tissues; and 5 (11.5%) samples of CRC tissues showed no expression. Based on these results, we confirmed that CIAPIN1 protein expression was lower in carcinomas tissues compared with adjacent normal tissues (Fig. 2(A)). We subsequently investigated whether the expression of CIAPIN1 correlated with the differentiation status and UICC stage as shown by immunohistochemistry. CIAPIN1expression was found to be increased in well-differentiated tumours (Fig. 2(B)). Furthermore, CIAPIN1 expression decreased gradually with increasing stage (Fig. 2(C)). This was consistent with the results of the immunohistochemistry experiments.

### CIAPIN1 expression increased in colon carcinoma cells after inducing differentiation

**Figure 3:**
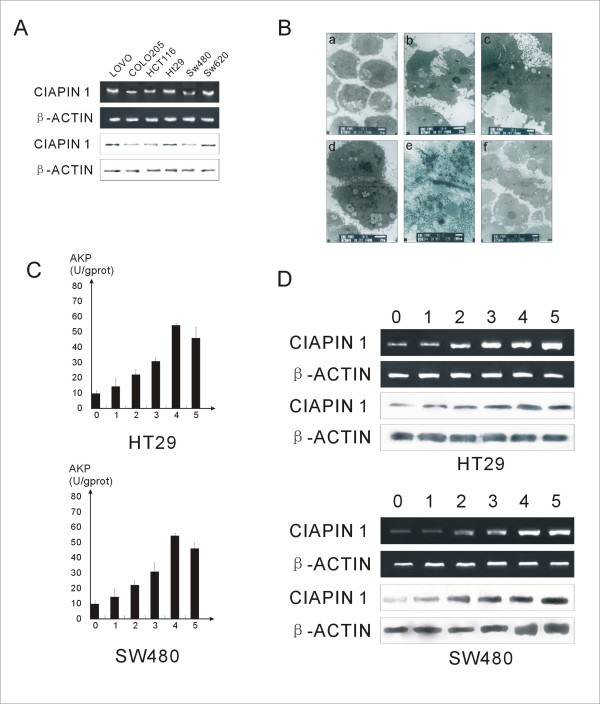
**(A) RT-PCR and western blotting results showing CIAPIN1 transcripts and protein in CRC cell lines**. β-Actin was used as an internal control. (B) Electron microscopy of colon cancer cells during the process of induced differentiation. (a) Undifferentiated colon cells; (b-f) Changes in ultra-microstructure during colon cell differentiation. There were special conformations such as cryptate structures, tight junctions and macula adherens between cells, as well as polarisation of microvilli in differentiated HT29 and SW480 cells. (C) AP level during induced cell differentiation in two different colon cancer cell lines. AP levels in HT29 and SW480 were measured from the 1st to the 5th day during the induced differentiation process and compared with untreated cells (day 0). (D) RT-PCR and Western blotting results showing CIAPIN1 transcripts and protein changes during HT29 and SW480 cell line differentiation from the 1st to 5th day of induced differentiation compared with untreated cells (day 0). β-Actin was used as an internal control.

CIAPIN1 gene and protein expression levels were tested in the human CRC cell lines LoVo, CoLo205, HCT116, HT-29, SW620, and SW480. As shown in Fig. 3A, CIAPIN1 expression was detected in all CRC cell lines. To investigate the expression of CIAPIN1 during CRC cell differentiation, in vitro studies with HT-29 and SW480 CRC cell lines were performed. Cells were treated with 2 mM sodium butyrate to induce differentiation. Following incubation, the differentiation status was confirmed by TEM examination of the presence of regular brush borders and tight junctions, which are structural markers of differentiation (Fig. 3B). In addition, butyrate-induced differentiation was measured by alkaline phosphatase (AP) activity. AP activity increased significantly in HT-29 and SW480 cells after 48 h of incubation (Fig. 3C). Both protein and mRNA expression levels of CIAPIN1 were measured in HT29 and SW480 cell lines at the indicated time points after butyrate treatment. Prolonged incubation with butyrate resulted in a time-dependent induction of CIAPIN1expression (Fig. 3D).

## Disscussion

CIAPIN1 was a recently reported anti-apoptotic molecule, but its biological function was far from being fully understood. In the previous study [12], we observed the distribution of the CIAPIN1 in a variety of cultured cell lines, including mouse fibroblast cell line NIH3T3, human tumor cell lines and immortalized cell lines by using immunofluorescence staining, immunohistochemistry and EGFP-CIAPIN1 fusion protein. It was shown that CIAPIN1 was distributed both in the cytoplasm, but it was more concentrated in the nuclei. To further confirm the above results, Western blot was used to detect the distribution of CIAPIN1 in the sub-cellular components by separating sub-cellular component of HepG2. The results were consistence with immunological method. It would indicate that CIAPIN1 might be involved in a cytoplasm-nucleus-nucleolus translocation process in the cells, and nucleoli might be the final area or one of the final areas where CIAPIN1 may play its biological function. Another possibility was that nucleoli might be an important storage place of CIAPIN1.

Our group found that CIAPIN1 protein existed in many organs of mice at different time points after birth. From 1 to 7 days after birth, the expression of CIAPIN1 gradually strengthened in heart, brain, liver, kidney and skeletal muscle [12]. The expression of CIAPIN1 could also be detected in many organs of the 5 month old human embryo. The expression level of CIAPIN1 in adult organs of endodermal and ectodermal origin was lower than that in 5 month old human embryo. But the result in the organs derived from mesoderm was the opposite [13]. These findings were suggested that CIAPIN1 was a new gene which was closely related to differentiation.

We previously confirmed that CIAPIN1 expression in gastric cancer, liver cancer and clear cell renal cell carcinoma (CCRCC) was down-regulated compared to their adjacent normal tissue counterparts. Furthermore, stable transfection of CIAPIN1 siRNA may contribute to carcinogenesis by accelerating cell proliferation and promoting cell-cycle progression in vitro and in vivo in the two tumour types [17,19-21]. Our group also demonstrated that CIAPIN1 inhibited gastric cancer and CCRCC cell proliferation, as well as cell-cycle progression by down-regulating CyclinD1 and up-regulating P27. CIAPIN1 siRNA could also significantly down-regulate the expression of Bcl-2, and up-regulate the expression of Bax but not that of PTEN in gastric cancer cells. These observations suggested that the siRNA constructs of CIAPIN1 could effectively down-regulate the expression of CIAPIN1 and reverse the multidrug resistant phenotype of gastric cancer cells.

In the present study, we first investigated the expression of CIAPIN1 in human CRC tissues and cell lines with the aim of investigating the molecular mechanisms of CRC tumorigenesis, and ultimately, to find a new molecular marker of the early diagnosis and prognosis of CRC. Our results showed that the CIAPIN1 protein was expressed in the nucleus and cytoplasm of colorectal glands epithelial cells, and CIAPIN1 expression was significantly decreased in CRC specimens compared to their adjacent non-cancerous tissues. The expression level of CIAPIN1 was closely related to the prognosis of patients with CRC. The overall survival of patients with high or moderate CIAPIN1 expression was significantly better than those with none or weak CIAPIN1 expression, and low expression levels of CIAPIN1 associated with the tumour stage, lymph node metastasis, distant organ metastasis and local recurrence were independent, poor prognostic factors of CRC. These data suggested that CIAPIN1 was associated with CRC, and the decreased expression of CIAPIN1 was correlated with a worse outcome of CRC patients. CIAPIN1 could be considered as a potentially valuable prognostic indicator in patients with CRC. This information might be useful to clinicians in providing individualized therapy for CRC patients with optimal benefit.

In this study, we also observed CIAPIN1 expression, at both the mRNA and protein levels, in the human CRC cell lines LoVo, CoLo205, HCT116, HT-29, SW620, and SW480. Our results suggest that CIAPIN1 gene expression might be different in CRC and normal colorectal tissue, and CIAPIN1 might be involved in colorectal carcinogenesis and development, as well as functioning as a tumor suppressor gene in CRC. To determine whether CIAPIN1 could be involved in the differentiation of CRC cells, we induced differentiation of HT29 and SW480 cells. We found that both the CIAPIN1 protein and mRNA levels increased gradually over time and with differentiation status. The changes in CIAPIN1 expression during the differentiation of CRC cells may provide an important clue to understand its function in CRC cell differentiation. This finding is consistent with Hao's data [12,13], but it still does not explain the function of CIAPIN1. More studies are needed to prove the regulatory mechanisms and signaling pathways of this gene in cell differentiation.

Previous study of our group had shown that CIAPIN1 was involved in the regulation of multidrug resistance (MDR) of gastric cancer cells [14,15,22]. We also found that the expression of CIAPIN1 was higher in the cells of gastric cancer SGC7901/VCR than in SGC7901 cells. In addition, it was demonstrated that CIAPIN1 could increase the expression of P-gp and the relative ratio of Bcl-2/Bax in SGC7901/VCR cells. CIAPIN1 siRNA could reverse the phenotype of MDR in SGC7901/VCR cells. These results suggested that inhibition of exocytosis of the anticancer drugs and resistance to chemotherapy-induced apoptosis might be the important mechanism for CIAPIN1 to mediate the MDR of gastric cancer cells. In the present study, we could not get direct evidence of CIAPIN1 regulating the MDR of CRC cells. But, we did find that CIAPIN1 played an important role in regulating the tumorigenesis and cell differentiation of CRC. There was no significant survival benefit for CRC patients from neoadjuvant chemotherapy. However, there was significant survival benefit from high expression of CIAPIN1.

Taken together, our results revealed that CIAPIN1 was differentially expressed in normal adjacent tissues and cancer tissues from the same CRC patient and that CIAPIN1 expression was closely related to the tumour state, local recurrence and UICC stage. Low expression level of CIAPIN1 might act as an independent, poor prognostic factor of CRC. These data suggested that CIAPIN1 might have an important role in the development of colorectal cancer. Although the regulatory mechanism of CIAPIN1 in CRC was still unclear, our studies had provided evidence that CIAPIN1 expression correlated with the differentiation level and prognosis of CRC, suggesting it was an important role in tumour biology.

## Competing interests

The authors declare that they have no competing interests.

## Authors' contributions

**HS, WW **and **QZ **designed the study and carried out the immunohistochemistry studies. **HL **and **SL **participated in western blot analysis and result interpretation. **YZ, XZ **and **HZ **prepared and provided the tumour biological samples and participated in the immunohistochemistry studies. **XL **and **NL **performed Quantitative analysis and participated in discussion. **CC **performed the statistical analysis and participated in the discussion. All authors read, discussed and approved the final manuscript.

## Pre-publication history

The pre-publication history for this paper can be accessed here:

http://www.biomedcentral.com/1471-2407/10/477/prepub
